# Toxicity of Functional Nano-Micro Zinc Oxide Tetrapods: Impact of Cell Culture Conditions, Cellular Age and Material Properties

**DOI:** 10.1371/journal.pone.0084983

**Published:** 2014-01-13

**Authors:** Heike Papavlassopoulos, Yogendra K. Mishra, Sören Kaps, Ingo Paulowicz, Ramzy Abdelaziz, Mady Elbahri, Edmund Maser, Rainer Adelung, Claudia Röhl

**Affiliations:** 1 Institute of Toxicology and Pharmacology for Natural Scientists, Christiana Albertina University Kiel, Kiel, Germany; 2 Functional Nanomaterials, Institute for Materials Science, Christiana Albertina University Kiel, Kiel, Germany; 3 Nanochemistry and Nanoengineering, Institute for Materials Science, Christiana Albertina University Kiel, Kiel, Germany; 4 Nanochemistry and Nanoengineering, Institute of Polymer Research, Helmholtz-Zentrum Geesthacht, Geesthacht, Germany; 5 ZEBET - Alternative Methods to Animal Experiments, Federal Institute for Risk Assessment, Berlin, Germany; University of California, Merced, United States of America

## Abstract

With increasing production and applications of nanostructured zinc oxide, e.g., for biomedical and consumer products, the question of safety is getting more and more important. Different morphologies of zinc oxide structures have been synthesized and accordingly investigated. In this study, we have particularly focused on nano-micro ZnO tetrapods (ZnO-T), because their large scale fabrication has been made possible by a newly introduced flame transport synthesis approach which will probably lead to several new applications. Moreover, ZnO-T provide a completely different morphology then classical spherical ZnO nanoparticles. To get a better understanding of parameters that affect the interactions between ZnO-T and mammalian cells, and thus their biocompatibility, we have examined the impact of cell culture conditions as well as of material properties on cytotoxicity. Our results demonstrate that the cell density of fibroblasts in culture along with their age, i.e., the number of preceding cell divisions, strongly affect the cytotoxic potency of ZnO-T. Concerning the material properties, the toxic potency of ZnO-T is found to be significantly lower than that of spherical ZnO nanoparticles. Furthermore, the morphology of the ZnO-T influenced cellular toxicity in contrast to surface charges modified by UV illumination or O_2_ treatment and to the material age. Finally, we have observed that direct contact between tetrapods and cells increases their toxicity compared to transwell culture models which allow only an indirect effect via released zinc ions. The results reveal several parameters that can be of importance for the assessment of ZnO-T toxicity in cell cultures and for particle development.

## Introduction

Nano-microstructures (NMS) of zinc oxide have been fabricated and studied in detail because of their multifunctional applications ranging from nanoscale electronic devices, lasers, sensors and significantly in biomedical engineering as consumer products [1]–[8]. The main advantages of ZnO include biocompatible nature, low costs availability and possible fabrications of its nanostructures by very simple growth processes. For example, due to their interesting antibacterial properties, ZnO nano-microstructures have served as promising prophylactic agents against bacterial infections [9]–[13]. ZnO structures of different size ranges as well as with complex shapes have been utilized for various biomedical applications but a detailed understanding about the caused effects by these structures is still open. Material properties like size, shape, method by which they have been synthesized etc. as well as cell culture conditions play equally important roles in determining the nanostructure's effect on cells. Synthesis by chemical routes involves different chemicals and thus obtained nanostructures exhibit chemically modified surfaces. In this regard, direct fabrication methods (e.g., physical vapour deposition, lithography techniques, etc.) are better as the obtained structures do not involve complex chemicals, however precise control over size, shape and cost-effectiveness are major issues. The ZnO tetrapods (ZnO-T) used in this work were synthesized by a recently introduced flame transport synthesis (FTS) approach [14], [15]. The main advantage of this technique is that it offers versatile synthesis of ZnO-T with dimensions ranging from nanoscale to microscale and large amounts (up to kilograms) can be easily synthesized in a very effective manner. The growth of these ZnO tetrapod structures has already been discussed earlier [15]. The arms of tetrapod exhibit hexagonal wurtzite crystal structure oriented along the c-axis with alternating Zn^2+^ and O^2−^ stacking planes. The tetrapod shape is very unique in the sense that it exhibits a three dimensional geometry with its four arms pointing along ∼109.5° angle with respect to each other. In reality, angles between tetrapod arms differ from perfect geometry to compensate the stresses induced by dislocations in the core of the seed ZnO particles [16]–[18]. These ZnO-T have already shown their potentials for several technological applications including their strong blocking capability of viral (herpes simplex virus type-1 and type- 2 (HSV-1 and HSV-2)) entry into the cells [19]–[22]. In presence of these ZnO-T, the viral entry into the cells is decreased as some of the viruses are trapped by ZnO-T. Illuminating these structures with UV light improved their virus trapping capability and therefore a further decrease in viral entry into the cells was observed [21], [22]. These ZnO-T exhibit oxygen vacancies which seem to be appropriate sites for herpes simplex virus attachment via hepran sulphate (HS) present in the virion envelope. Trapping of HSV-1 and HSV-2 by ZnO tetrapods has been shown to prevent HSV-1 and HSV-2 infections *in-vitro*. Thereby, ZnO-T seem to be very promising prophylactic agents for the prevention of HSV-1 and HSV-2 infection *in-vivo* [21]–[23]. Therefore cytocompatibility of these ZnO-T in this context and for other biomedical applications in general is a very important issue which still requires to be investigated in detail.

In order to get a better understanding of the parameters that have an impact on toxicity assessment of ZnO-T *in-vitro* we have focused on the detailed analysis of various cell culture conditions as well as different material properties which could affect the biocompatibility of these submicron sized ZnO-T for human dermal fibroblasts (NHDF) as target cells. In this study, we have considered various cell culture conditions as well as different material properties. Important cell culture conditions that might influence the biological effect of ZnO-T are cell density, stage of cell growth, age of the cells and cell to cell contacts. Cytotoxicity studies of different ZnO nanoscale structures with respect to various cells have been performed [13], [24]–[28]. Heng et al. have described that the particle to cell ratio plays a crucial role for particle's toxicity [26]. Furthermore, it has been shown that the cytotoxic effect of ZnO nanoparticles (ZnO NP) also depends upon the proliferation rate of mammalian cells and that ZnO NPs exhibit a selective lethal effect on rapidly proliferating cells [13], [27]. Due to these effects, ZnO NPs also received much attention for their implications in cancer therapy [28]. To the best of our knowledge, no studies exist so far which have examined the impact of cell line age, i.e., the number of preceding subcultures/passages on the toxicity of ZnO NPs or tetrapods [29]. Material properties that might impact the cytocompatibility include, e.g., particle size, shape, defects and surface reactivity. Hanley et al. observed that smaller ZnO NPs are more toxic to mammalian cells than larger ZnO NPs [25] and also the shape of the nanostructures has strong influence on cytotoxicity [30]. The existence of inherent defects such as O vacancies or Zn interstitials further increase different functionalities of these ZnO-T and this mainly depends upon the experimental conditions and the used synthesis technique. Since ZnO exhibits a direct bandgap of around 3.37 eV, the oxygen vacancy content in ZnO-T can be easily increased (by illuminating them with UV light) or decreased (by annealing them at moderate temperatures in oxygen rich environment). Thus ZnO structures exhibit unique advantage of changing their surface reactivities (from hydrophobic to hydrophilic and vice versa) without physically destroying them [31], [32]. The surface reactivity can significantly influence the biological efficacy of these ZnO structures, e.g., the viral trapping capability of UV illuminated ZnO-T is higher than that of normal (un-illuminated) ZnO-T as observed in HSV cell entry studies [21], [22]. Other studies have shown that cytotoxicity is associated with accumulation of ZnO NPs inside cells and breakage of cell- and mitochondria membranes, increasing micronuclei [33]–[36] and disturbing zinc ion and/or calcium homeostasis [37]–[39].

Due to the large biologically effective diameter, our ZnO-T structures cannot be taken up by fibroblasts. This is of great advantage as we can rule out that toxicity might be a consequence of cellular uptake, while we continue to have nanospecific material properties due to the dimension of the tetrapod tips. Some studies have shown that release of Zn^2+^ ions in the culture medium or inside cells causes significant cell toxicity [39]–[42]. In contrast, other reports have demonstrated that the particulate matter in contact with cells leads to cell death [28], [43]. To investigate the cellular mechanisms of ZnO-T toxicity, the role of direct cell contact and dissolved Zn^2+^ ions has also been examined in this study. A detailed understanding of the impact of material properties, biological conditions and the involved mechanisms leading to toxicity will be of high interest for the toxicological assessment of other metal oxide nano-microstructures and it will also help the biomaterials community to consider possible toxicological effects for further development of ZnO nano-microstructures.

## Results

### A) Particle dependent effects

#### A1. Roles of ZnO tetrapod's age and concentration on cytotoxicity

**Figure 1:**
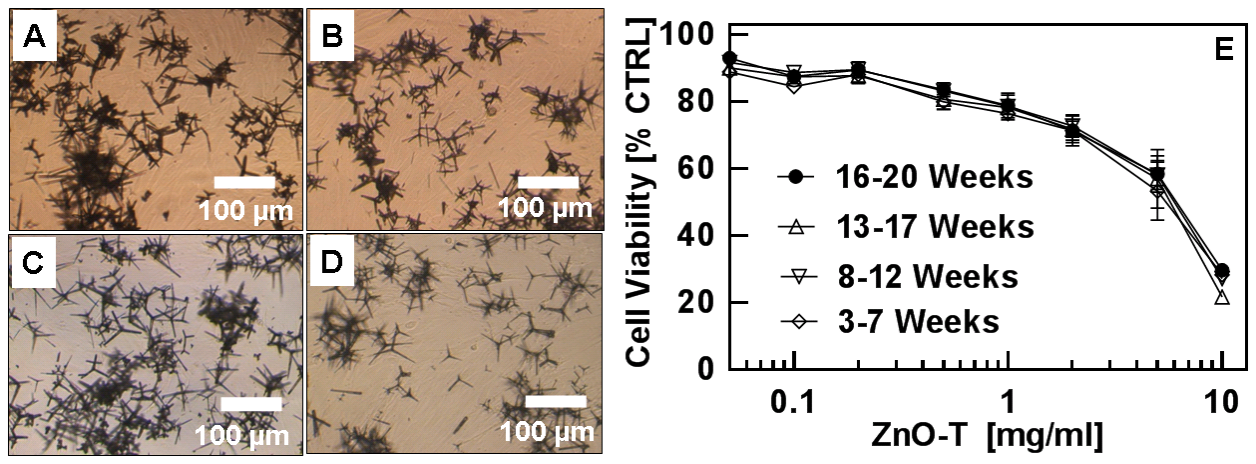
Morphology (A–D) and cytotoxicity (E) of ZnO-T structures synthesized at different dates. Ages of ZnO-T used for cell treatment were: A = 16–20 weeks, B = 13–17 weeks, C = 8–12 weeks and D = 3–7 weeks. Passage number of normal human dermal fibroblasts (NHDF): P7–P11. Seeded cell number: 50000 cells/cm^2^; ZnO-T concentration: 0.05–10 mg/ml; time prior treatment: 48 h; duration of treatment: 24 h. [Each symbol represents the mean ± SE of n = 3–5 independent experiments with fourfold determinations. CTRL is short abbreviation for control].

For applications of nanostructures in general, it is desirable that they retain their physical and chemical characteristics for longer durations without any specific storage requirement. But for nanostructures it is a very challenging task as they are quite sensitive to the local environment and hence are always stored in prescribed conditions, e.g., in vacuum, covered with appropriate capping agents etc. In order to investigate whether cytotoxic behaviour of ZnO-T is affected with respect to time, ZnO structures synthesized at different time intervals have been used for study. These ZnO-T were synthesized 16, 13, 8 and 3 weeks before their utilization in the first experiments with cells. The time span between the first and the last experiments was 4 weeks. It has been observed that physical appearance of these ZnO-T does not change even after 2 years but since the bandgap is UV sensitive, the structures were always stored in a dark place and at room temperature. The mean EC_50_ value for the reference (control) specimen is 6.1±0.6 mg/ml and with increasing ZnO-T concentrations, the cell viability decreases. As shown in Figure 1E, concentration-effect curves of all four samples (prepared at different time intervals), with concentrations up to 10 mg/ml over incubation period of 24 h, are approximately same. Mean EC_50_-values vary between 6.1±0.6 mg/ml for the oldest and 5.5±0.5 mg/ml for the youngest sample (Tab. 1).

**Table 1:** Impact of age of ZnO nano-microtetrapods on cell toxicity.

ZnO-T [Age]	EC_50_±SE [mg/ml]
16–20 Weeks	6.1±0.6
13–17 Weeks	5.7±0.6
8–12 Weeks	6.0±0.2
3–7 Weeks	5.5±0.5

#### A2. Influence of surface charge of ZnO tetrapods on cytotoxicity

**Figure 2:**
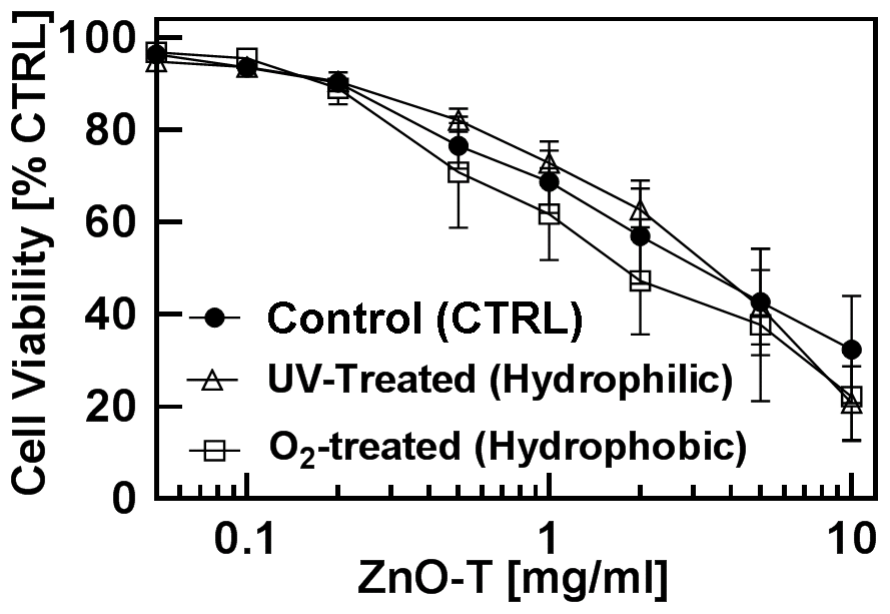
Cytotoxic effect of ZnO-T with different surface charges. Cytotoxicity of untreated or either pre-treated ZnO-T with O_2_ or UV-light. Cell viability of normal human dermal fibroblasts (NHDF) was determined by the MTT assay. Passage number of fibroblasts: P8–P20. Seeded cell number: 50000 cells/cm^2^ ZnO-T concentration: 0.05–10 mg/ml; time prior treatment: 48 h; duration of treatment: 24 h. Each symbol represents the mean ± SE of at least n = 3–5 independent experiments with fourfold determinations.

In order to get the information about the impact of ZnO-T′s surface charge on cytotoxicity, fibroblasts were treated with different concentrations of UV illuminated (hydrophilic) and oxygenated (hydrophobic) ZnO-T and cytotoxicities were compared with untreated tetrapods. As shown in Figure 2, neither UV illumination nor O_2_-treatment on ZnO-Ts led to any significant change in cell viability of NHDF after 24 h of treatment (Tab. 2).

**Table 2:** Impact of surface charge on cell toxicity.

ZnO-T	EC_50_±SE [mg/ml]
Untreated	5.3±0.7
O_2_-treated (hydrophobic)	4.5±0.4
UV-treated (hydrophilic)	3.6±0.7

#### A3. Dependence of ZnO tetrapod morphologies on cytotoxicity

**Figure 3:**
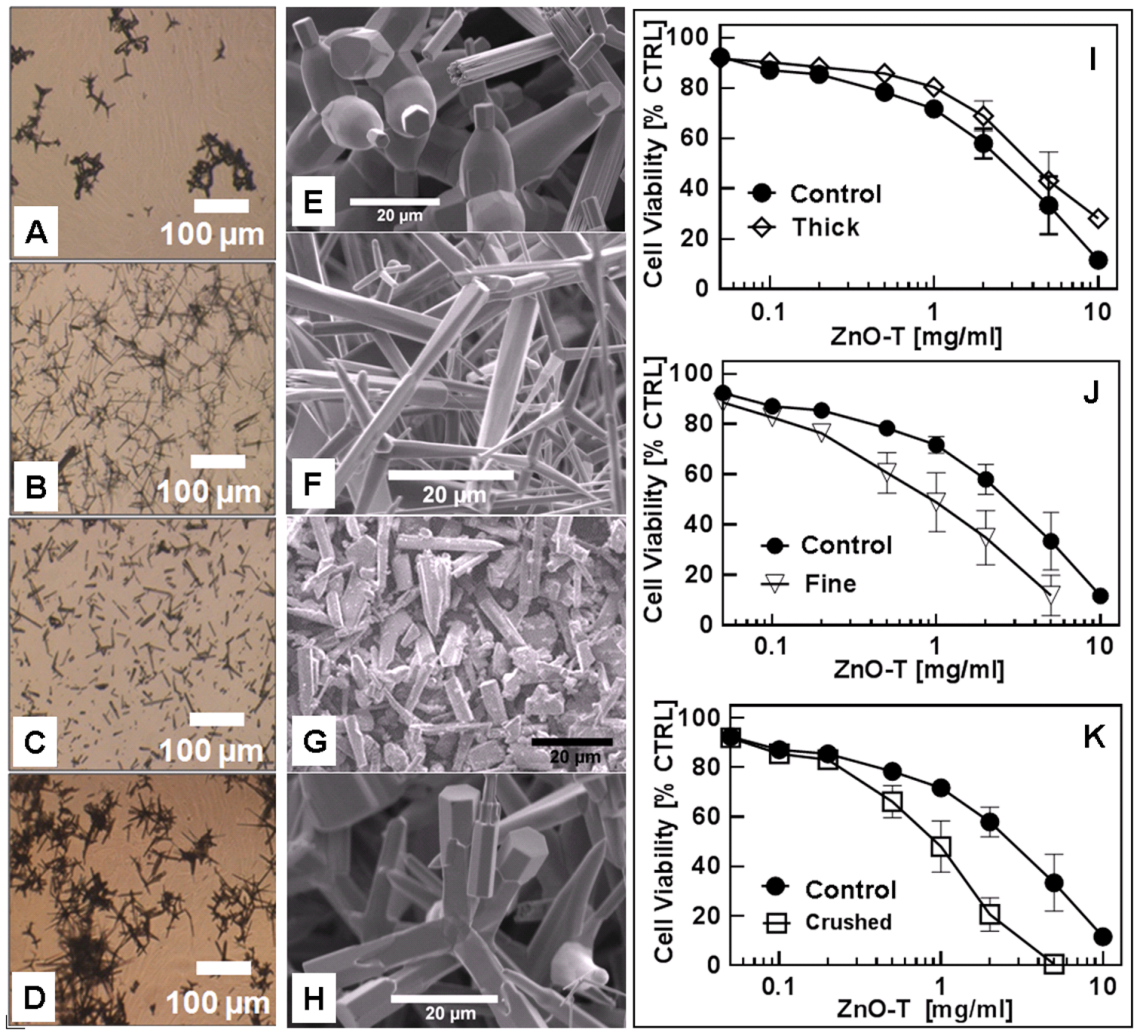
Effect of ZnO-T morphology on cytotoxicity: Phase contrast microscopy (A–D) and scanning electron microscopy (SEM) (E–H). A, E: ZnO-T “thick”; B, F: ZnO-T “fine”; C, G: ZnO-T reference “crushed”; D, H: ZnO-T “reference”, (I–K) Viability (MTT-assay) of treated human dermal fibroblasts (NHDF). Passage number of NHDF: P10–P14. Seeded cell number: 50000 cells/cm^2^; ZnO-T concentration: 0.05–10 mg/ml; time prior treatment: 48 h; duration of treatment: 24 h. Each symbol represents the mean ± SE of n = 3–5 independent experiments with fourfold determinations.

**Figure 4:**
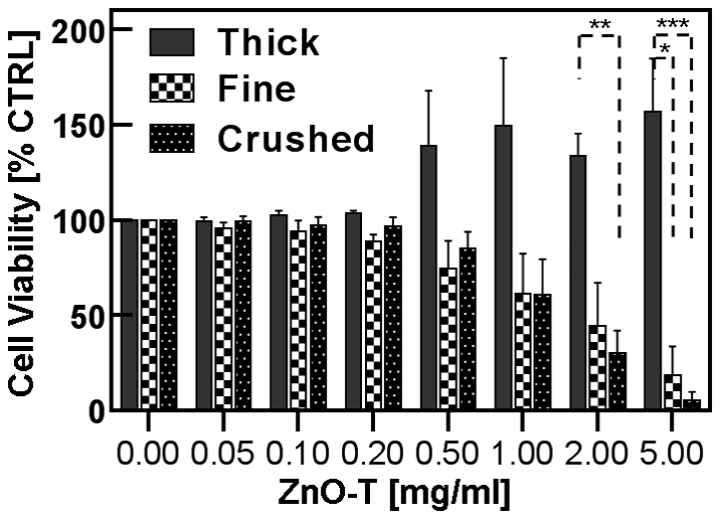
Percentage increase or decrease of cytotoxicity depending on size/shape of ZnO-T compared to the reference probe (ZnO-T reference) at each concentration [% viable cells of probe/% viable cells of reference * 100]. Each value shown is the mean of three independent experiments (± SE. Statistical significance determined by one way analysis of variance: thick vs. crushed 2 mg/ml (** = p<0.01); thick vs. fine 5 mg/ml (* = p<0.05); thick vs. crushed 5 mg/ml (*** = p<0.001) (post hoc test: Bonferroni).

During synthesis, different shapes of ZnO-T are achieved as shown in Figure 3 (A–H). To get insight into the impact of these structural changes on the cytotoxicity, different types of ZnO-T structures (long and thin, short and thick) have been selected for experiment with cells. Additionally, crushed ZnO-T which exhibit rod like structures, were also used and compared with ZnO-T (reference). As a result, the total surface area of smaller structures and thinner tetrapods per unit mass increased in comparison to that of compact (thicker) tetrapods (Figure 3I-K, 4). Due to crushing, the arms of tetrapods get broken which leads in an increase in resultant surface area. The surfaces of the broken ends of tetrapod's arms are very rough with several sharp kinks. These kinks can easily penetrate into the cell walls and also the possibility of higher Zn ions release from these broken surfaces could strongly affect their cytotoxic behaviour. The MTT assay revealed a higher cytotoxicity of crushed and thinner tetrapods after 24 h incubation. The mean EC_50_ values for crushed and fine tetrapods were found to be approximately 60% lower than that of thick tetrapods which is about 40% higher than the EC_50_ value corresponding to reference tetrapods (Tab. 3). Significant differences in cytotoxicities arose at 2 mg/ml ZnO between the thick and crushed and could be seen at 5 mg/ml ZnO between the thick and the crushed as well as between the thick and fine tetrapods (Figure 4).

**Table 3:** Impact of morphology on cell toxicity.

ZnO-T	EC_50_±SE [mg/ml]
Reference (CTRL)	3.2±0.3
Crushed	1.0±0.1
Fine	1.2±0.2
Thick	4.5±0.4

### B) Cell culture dependent effects

#### B1. Effect of cell passage number on ZnO tetrapod's cytotoxicity

**Figure 5:**
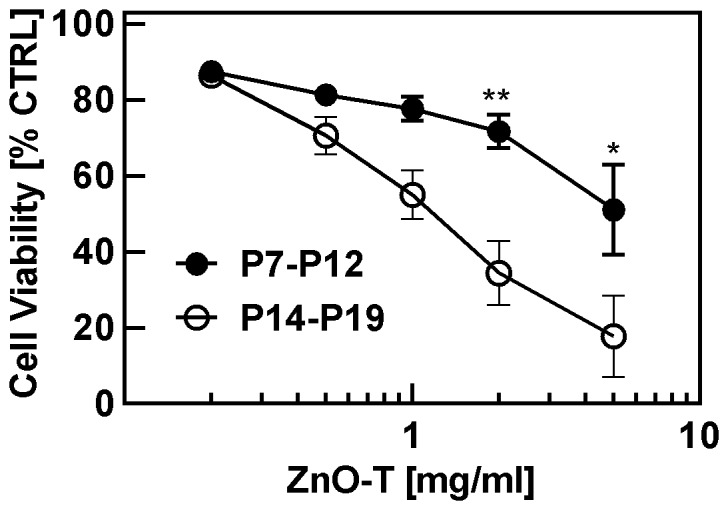
Effect of ZnO-T on cell viability determined by the MTT-assay depending on passage number of normal human dermal fibroblasts (NHDF). Seeded cell number: 50000 cells/cm^2^; ZnO-T concentration: 0.2–5 mg/ml; time prior treatment: 48 h; duration of treatment: 24 h. Each symbol represents the mean ± SE of n = 3 independent experiments. Statistical significance determined by one way analysis of variance: P7–P12 vs. P14–P19 2 mg/ml (** = p<0.01) and 5 mg/ml (* = p<0.05).

In the course of our experiments we have observed large variations in cytotoxicity of ZnO-T with respect to NHDF cells and therefore it is very important to investigate the role of various cell culture conditions which has been investigated here. First we examined whether there is any difference between using fibroblasts of a low or a high passage numbers. Cells of earlier passages have been more resistant to ZnO-T than those of later passages as demonstrated in Figure 5. NHDF with low passage numbers displayed, e.g., a cell viability of 34%±8% (median ± SE) at a concentration of 2 mg/ml ZnO-T whereas NHDF at high passage numbers exhibited a cell viability of 72%±4% (median ± SE) at the corresponding concentration. Mean EC_50_ values are shown in Tab. 4.

**Table 4:** Impact of passage number on cell toxicity.

ZnO-T	EC_50_±SE [mg/ml]
P7–P12	>5
P14–P19	1.4±0.2

#### B2. Impact of different cell seeding densities on the toxicity of ZnO tetrapods

**Figure 6:**
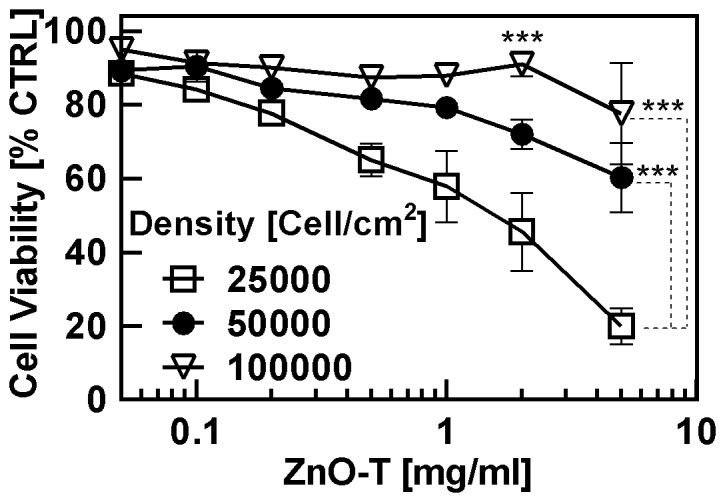
Effect of cell density on ZnO-T cytotoxicity. Cell viability of normal human dermal fibroblasts (NHDF) was determined by the MTT assay. Passage number: P11–P15. Seeded cell numbers: 25000 cells/cm^2^, 50000 ells/cm^2^ and 100000 cells/cm^2^; ZnO-T concentration: 0.05–5 mg/ml; time prior treatment: 48 h; duration of treatment: 24 h. Each symbol represents the mean ± SE of n = 3 independent experiments with fourfold determinations. Statistical significance determined by one way analysis of variance: 25000 vs. 50000 cells/cm^2^ 2 mg/ml (*** = p<0.001) and 5 mg/ml (*** = p<0.001); 100,000 vs. 50000 cells/cm^2^ and 5 mg/ml (*** = p<0.001).

Variation in cytotoxicity of ZnO-T with respect to different NHDF cell densities is shown in Figure 6. It is evident that the cytotoxicity of ZnO-T also depends on the cell density. The higher the cell density, the larger is the cell viability with respect to concentration of ZnO-T. The EC_50_ values revealed an increasing sensitivity of fibroblast cells towards ZnO-T with decreasing cell density (Tab. 5).

**Table 5:** Impact of seeding density on cell toxicity.

[Cells/cm^2^]	EC_50_±SE [mg/ml]
25000	1.9±0.3
50000	6±0.7
100000	>10

#### B3. Effect of the cell density on cytotoxicities of ZnO tetrapods and ZnCl_2_ depending on the number of preceding culture passages

**Figure 7:**
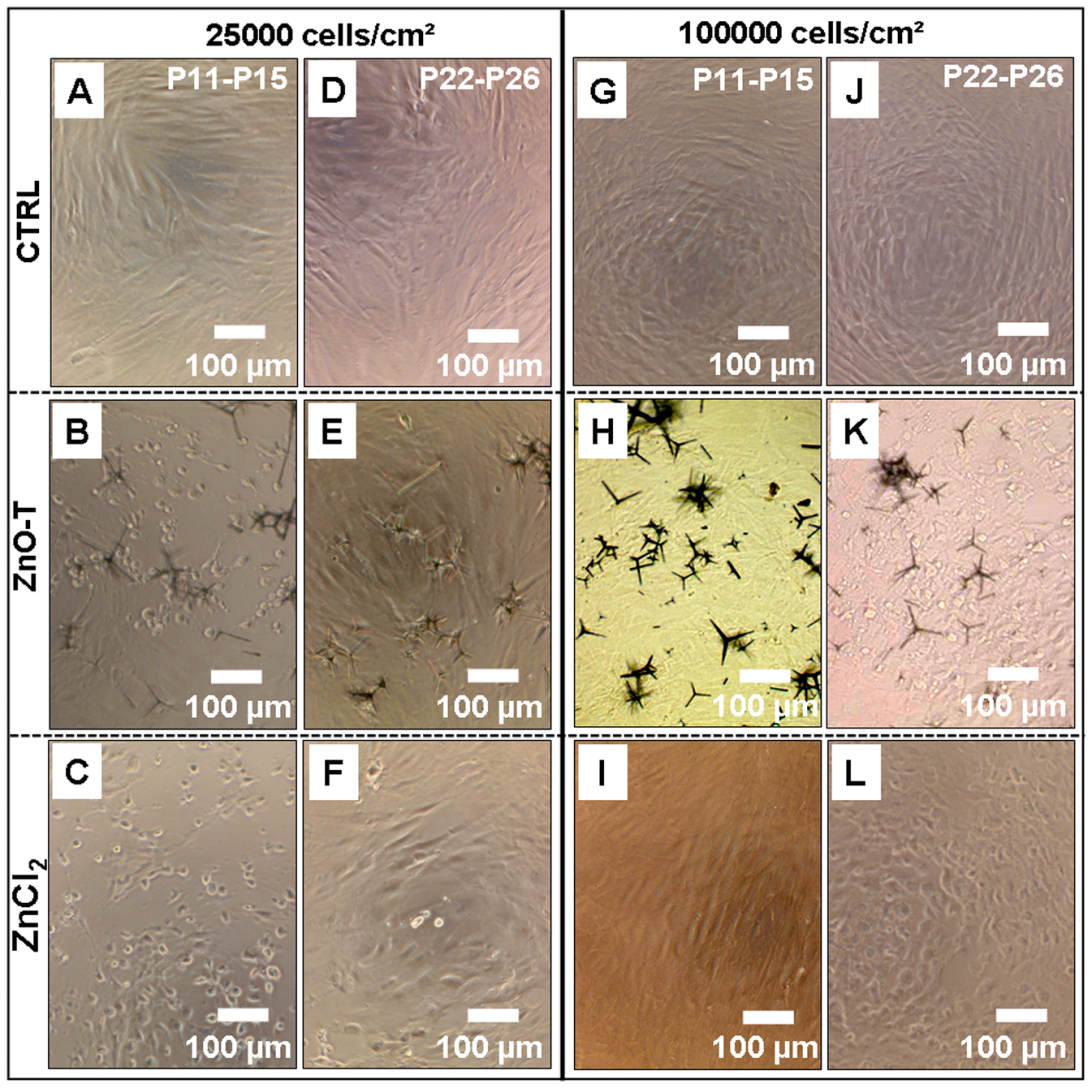
Phase contrast images of normal human dermal fibroblasts (NHDF) depending on passage numbers and seeding cell density after 24 h treatment with ZnO-T and ZnCl_2_. A, D, G and J: untreated NHDF; B, E, H and K: 0.5 mg/ml ZnO-T; C, F, I and L: 30 µg/ml ZnCl_2_. Passage numbers: P11–P15 (A–C and G–I) vs. P22–P26 (D–F and J–L). Seeded cell number: 25000 (A–F) vs. 100000 cells/cm^2^ (G–L); time prior treatment: 48 h; duration of treatment: 24 h. Each symbol represents the mean ± SE of n = 3 independent experiments with fourfold determinations. Additionally experiments were done with three different frozen stocks of NHDF to verify the result.

**Figure 8:**
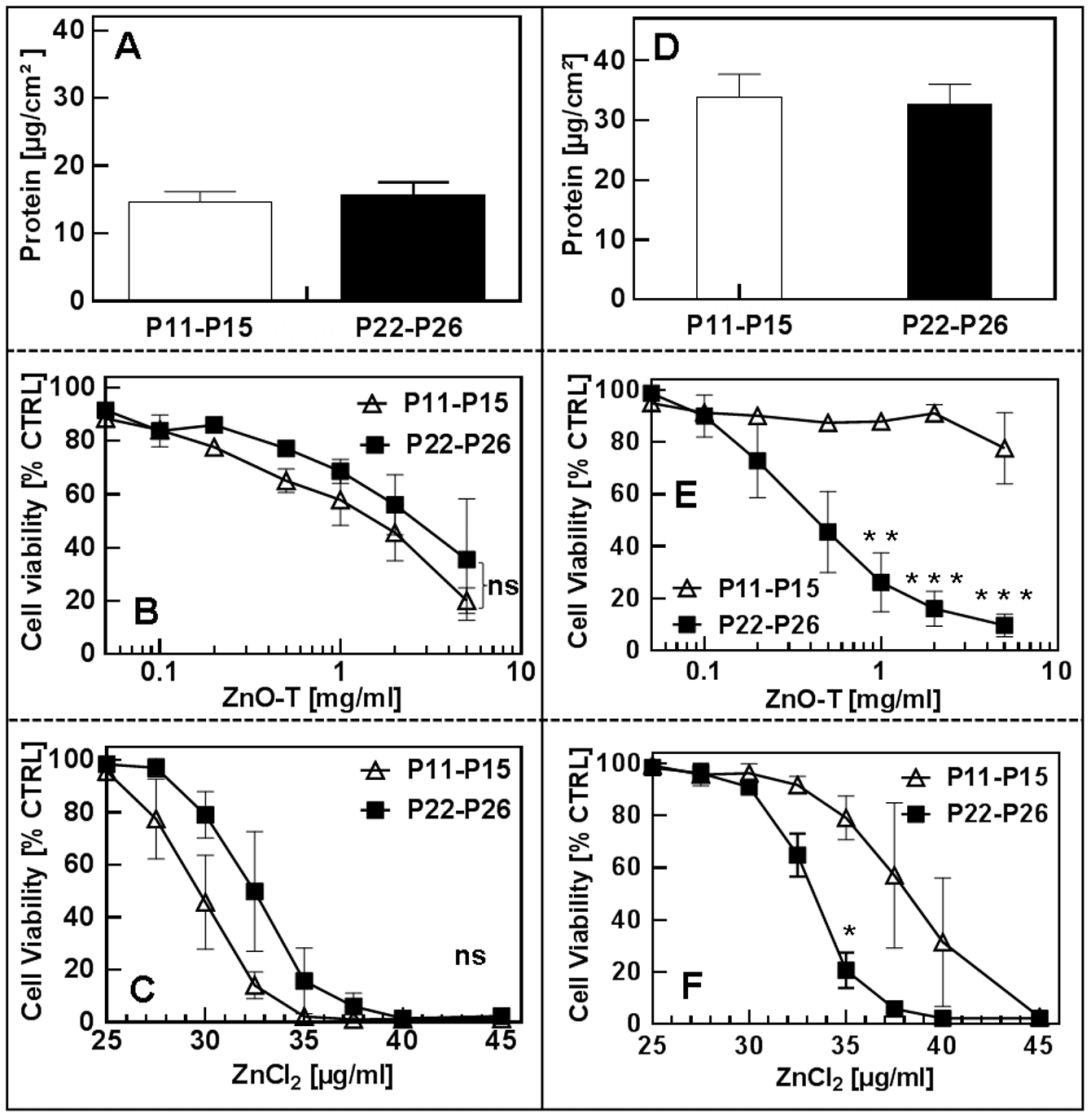
Effect of passage number and seeding cell density of NHDF on ZnO-T and ZnCl_2_ cytotoxicity. A and D: cell protein concentrations of controls determined by the Lowry-assay. Passage numbers: P11–P15 vs. P22–P26. Seeded cell numbers: 25000 (A–C) vs. 100000 cells/cm^2^ (D–F); ZnO-T concentration: 0.05–5 mg/ml; ZnCl_2_ concentration: 25–45 µg/ml; time prior treatment: 48 h; duration of treatment: 24 h. Each symbol represents the mean ± SE of n = 3 independent experiments with fourfold determinations. Additionally experiments were done with three different frozen stocks of NHDF to verify the result. Statistical significance determined by one way analysis of variance: cell density: 100000 cells/cm^2^, ZnO-T: P11–P15 vs. P22–P26 1 mg/ml (** = p<0.01), 2 mg/ml (*** = p<0.001) and 5 mg/ml (*** = p<0.001). Cell density: 100000 cells/cm^2^, ZnCl_2_: P11–P15 vs. P22–P26 35 µg/ml (* = p<0.05).

Since both parameters, passage number and cell seeding density individually play an important role for ZnO-T′s cell biocompatibility, we have performed further experiments in this context. In Figures 7 and 8 cytotoxic effects of ZnO-T and ZnCl_2_ on NHDF with low (P11–P15) and high (P22–P26) passage number depending on cell density are shown. The cytotoxicity of ZnO-T on NHDF with either high or low passage numbers is found to be similar when determined at a low cell density (25000 cells/cm^2^) as demonstrated in Figure 8. Nevertheless, at a high seeding cell density of 100000 cells/cm^2^ toxicity on cells of higher passage number (P22–P26) has increased while that on cells of lower cell passage number (P11–P15) significantly decreased (Figure 8). A similar tendency for cell viability could be observed in cell cultures treated with ZnCl_2_ (Figure 8). Differences in cell density due to a different proliferation rate as reason for the differing toxic potential have been excluded by measuring protein contents in control cultures (Figures 8A and 8D). Mean EC_50_ values depending on cell seeding density and passage number are shown in Tab. 6.

**Table 6:** Impact of seeding density + fibroblast passage number on cell toxicity.

[Cells/cm^2^]	ZnO-T EC_50_±SE [mg/ml]/Zn [mM]		ZnCl_2_ EC_50_±SE [g/ml]/Zn [mM]	
	P11–P15	P22–P26	P11–P15	P22–P26
**25000**	1.9±0.3/18.8±3	3.3±0.5/32.6±5	29.7±0.4/0.1±0.001	32.4±0.4/0.11±0.001
**100000**	>10/>98.7	0.6±0.1/5.9±1	38.0±0.7/0.13±0.002	33.3±0.2/0.12±0.001

#### B4 Comparison of cytotoxicities of ZnCl_2_, spherical ZnO NP and ZnO tetrapods

**Figure 9:**
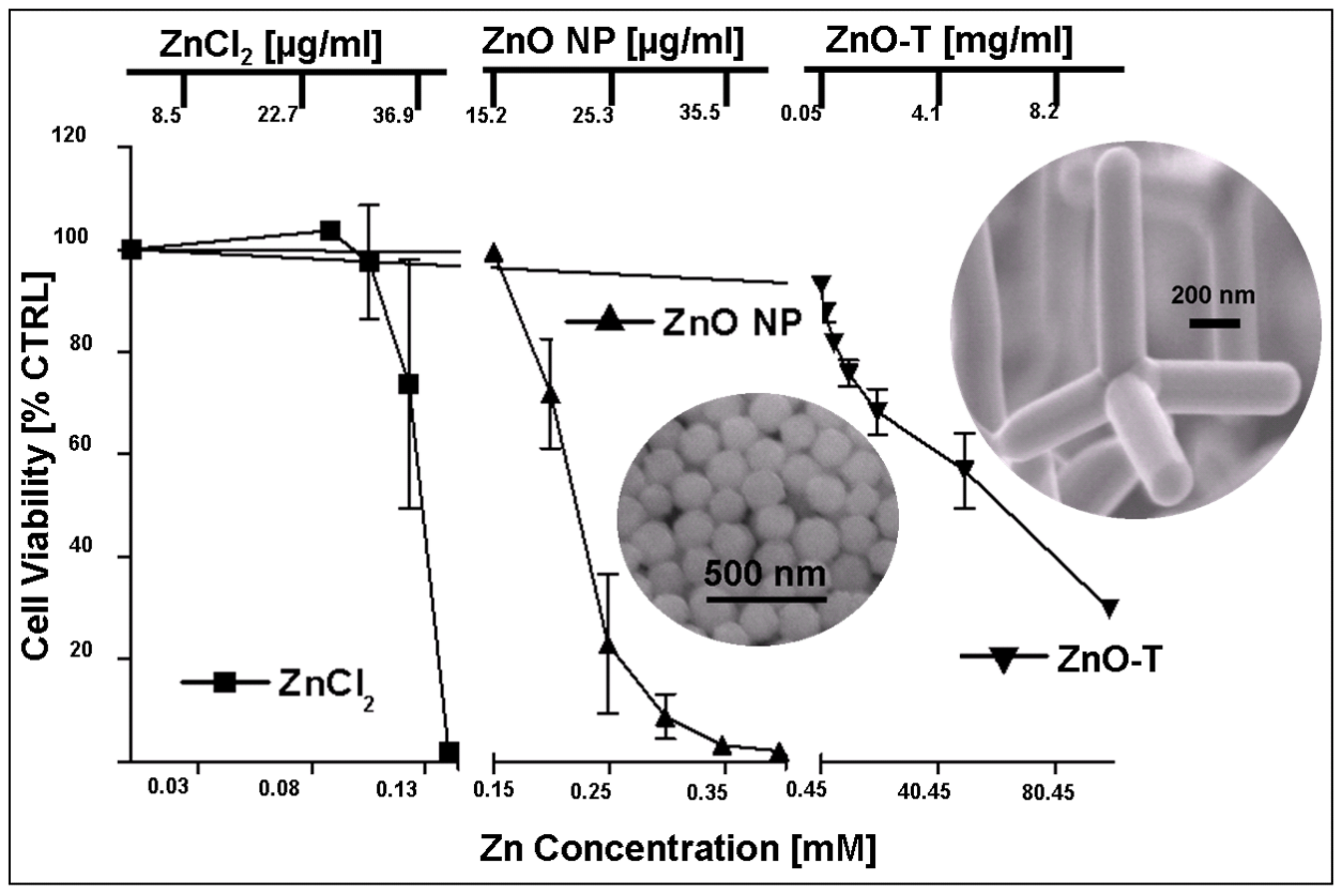
Comparing the cytotoxic effect of ZnCl_2_, spherical ZnO NP and ZnO-T on normal human dermal fibroblasts (NHDF) determined by the MTT-assay. Zn content is calculated in [mM] according to the used probe concentration. Passage number: P6–P9. Seeded cell number: 50000 cells/cm^2^; time prior treatment: 48 h; duration of treatment: 24 h. Each symbol represents the mean ± SE of n = 3 independent experiments with fourfold determinations. Statistical significance determined by one way analysis of variance: EC_50_ values are highly significant (p<0,001) different (Table 7). The typical SEM images of spherical ZnO NP (average diameter is ∼ 150 nm) and ZnO are shown as inset images which were used for investigation in present case.

In Figure 9 the cytotoxic effects of ZnCl_2_, ZnO NP and ZnO tetrapods on NHDF cells are shown depending on their Zn concentrations in mM. The determined viability of NHDF cells after 24 h treatment shows a significantly lower cytotoxicity of ZnO tetrapods (EC_50_: 6.1±0.6 mg/ml ZnO, i.e., 60.2±6.0 mM zinc) compared to ZnCl_2_ (EC_50_: 0.036±0.0014 mg/ml ZnCl_2_, i.e., 0.13±0.01 zinc) and to spherical ZnO nanoparticles (EC_50_: 0.022±0.001 mg/ml ZnO, i.e., 0.22±0.01 mM zinc) (Tab. 7). The cytotoxicity of ZnO increases with an increase in surface area. Ionic zinc causes the highest cytotoxic effect. Therefore, the potential to release zinc ions seems to play an important role in cytotoxicity.

**Table 7:** EC_50_ values for ZnCl_2_, spherical ZnO NP and ZnO-T.

[Cells/cm^2^]	EC_50_±standard error	
	in mg/ml	in Zn^2+^ mM
**ZnCl_2_**	0.036± 0.0014	0.13±0.01
**ZnO NPs**	0.022±0.001	0.22±0.01
**ZnO-T**	6.1±0.6	60.2±6.0

#### B5. Role of cell contact for ZnO tetrapods cytotoxicity

**Figure 10:**
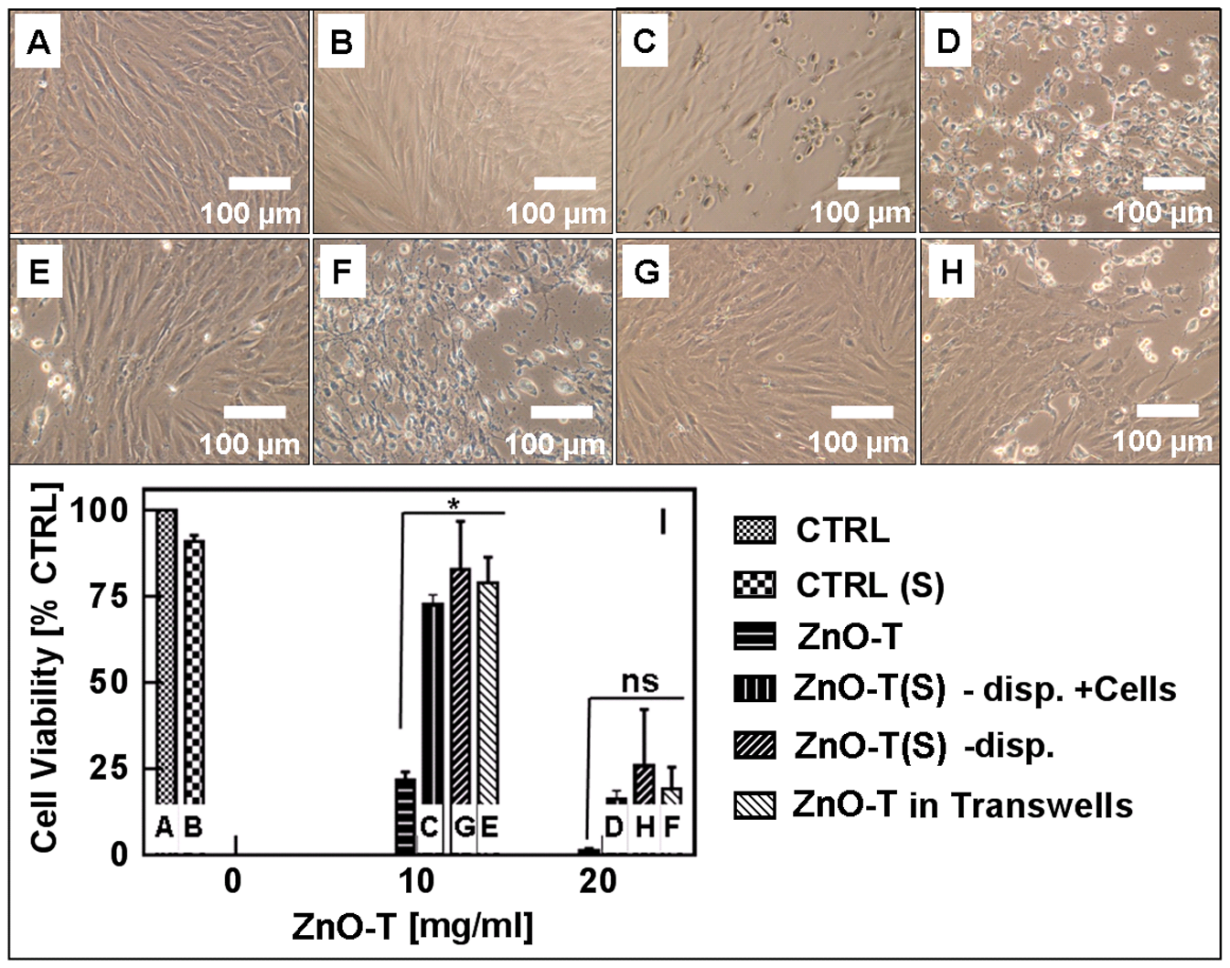
Role of direct cell contact for ZnO-T toxicity shown by phase contrast microscopy and changes in cell viability (MTT-Assay). Column diagram shows the cytotoxicological effects of different probes on normal human dermal fibroblasts (NHDF). CTRL (Image: A) = cell culture medium as control; CTRL (S) (Image: B) = supernatant of cell culture medium 24 h pre-incubated on NHDF as control; ZnO-T = ZnO-T in dispersion directly on NHDF (10 mg/ml or 20 mg/ml (Images: not shown due to overlapping of ZnO tetrapods layer)); ZnO-T (S) – disp. + cells = supernatant of ZnO-T in culture medium pre-incubated on NHDF for 24 h (10 mg/ml (Image: C) or 20 mg/ml (Image: D)); ZnO-T (S) – disp. = supernatant of ZnO-T in culture medium pre-incubated for 24 h without NHDF (10 mg/ml (Image: G) or 20 mg/ml (Image H); ZnO-T in Transwells = ZnO-T in dispersion (10 mg/ml (Image: E) or 20 mg/ml (Image: F) into transwells to prevent cell contact. Each value shown is the mean of three independent experiments (± SE). Passage number: P12–P14. Seeded cell number: 50000 cells/cm^2^; time prior treatment: 48 h; duration of treatment: 24 h. Each column represent the mean ± SE of n = 3 independent experiments. Statistical significance determined by one way analysis of variance: ZnO-T vs. C/D, G/H and E/F (* = p<0.05, ns = not significant).

The higher toxicity of ZnCl_2_ and spherical ZnO nanoparticles (ZnO NP) compared to ZnO-T indicates that release of Zn^2+^ ions also plays an important role for the toxic potency of zinc oxide. Cell culture experiments were performed in order to examine up to which extent zinc ion release might play a role in ZnO tetrapods induced toxicity. These cell culture experiments were carried out only with indirect cell contact via cell culture medium by using culture plate well inserts/transwells (conditioned culture medium) and the corresponding results are shown in Figure 10. We performed experiments with supernatant of ZnO-T (20 mg/ml and 10 mg/ml) pre-incubated for 24 h with or without the presence of fibroblasts. To rule out that a decrease in cell viability might only be due to the loss of nutrients in the medium caused by pre-incubation, we used medium pre-incubated on cells for 24 h without ZnO-T as a control (Figure 10). Additionally, ZnO-T were added on the one hand directly to fibroblasts and on the other hand into transwells positioned in fibroblast wells, so that a direct cell contact to ZnO-T was not possible. As shown in Figure 10 (diagram) ZnO-T dispersions of 10 mg/ml and 20 mg/ml resulted in dramatic decrease of cell viability as expected from the preceding experiments. Toxicity of ZnO-T at 10mg/ml with no direct contact to the cells was in contrast almost absent. It also appeared at 20mg/ml though to a lesser extent than in experiments with direct cell contact. Presence of fibroblasts during conditioning of medium did not change the toxicity of ZnO-T.

## Discussion

The application of nanoscale materials in food and consumer products as well as in medical devices requires the implementation of detailed cytotoxicological investigations for better consumer safety. ZnO nano-microtetrapods utilized in this study are able to bind HSV-1, HSV-2 and thus could serve as active agents on the skin to prevent herpes infection [21]–[23]. Due to the fact that HSV-1 infection causes skin lesions characterized by vesicles, cytotoxicological investigations in this study were carried out with NHDF, as fibroblasts exhibit a central role in the process of wound healing and in maintaining the skin integrity [44]–[46]. In the course of our experiments with ZnO-T, we have observed major varieties in their cytotoxic behaviour. These findings led to the presumption that changes of either material or cell properties might have been responsible for the variable results. Experiments concerning the properties of ZnO-T included analyses with regard to age and shape/size of ZnO structures. In addition to this, we have also investigated whether different surface reactivities induced by UV illumination or O_2_ treatment might affect cytotoxicity.

The observed results indicate that the aging of the ZnO-T has almost no influence on cytotoxicity (Figure 1E). During the storage of ZnO-T the UV light or atmospheric oxygen causes variations in oxygen vacancies and thus their hydrophilicity would change [23]. This again could change their cytotoxic behaviour, nevertheless, we have not observed any significant differences of UV-illuminated or oxygenated ZnO-T in our experiment. Even pre-treatment of ZnO-T with either UV light or O_2_ overnight showed no influence on cytotoxicity for NHDF (Figure 2), although other studies have shown that treatment of ZnO-T in this way could change their capability to bind virus particles [21], [22]. Zhang et al., have demonstrated the influence of surface charge on cytotoxicity [47]. The more positive the surface charges of ZnO NP, the higher the cytotoxic effect is which can be understood in terms of the static attraction between negatively charged cells and positively charged ZnO nanoparticles. Following this assumption, UV-treated ZnO-T should be less toxic due to increased number of oxygen vacancies, leading to repulsion between tetrapod's surface and cells. On the other hand, UV treated ZnO-T become more hydrophilic which increases their solubility and bioavailability. O_2_-treatment of ZnO-T results in passivation of oxygen vacancies which switches the wetting behaviour of these ZnO-tetrapods from hydrophilic to hydrophobic [23]. O_2_ saturation neutralizes negative surface charges of ZnO-T by which the interaction between ZnO-T and negatively charged cell membrane might increase leading to enhanced toxicity. On the other hand availability decreases due to the lower solubility of neutralized ZnO-T. In our studies no significant changes in cytotoxicity occurred after O_2_ - or UV- light treatment of ZnO structures. Zhang et al. described a cell line-dependent quite low (up to 30%) effect of changed surface charge of ZnO NP (achieved by coating with different concentrations of polyacrylic acid) on cytotoxicity [47]. In our study, we compared the cytotoxicities of negatively charged and neutralized ZnO-T and no significant change in toxicity is observed. It is difficult to rule out because the applied cell culture system is not sensitive enough to this kind of surface changes. In our case it seems that irrespective of surface charges (reference, oxygenated or UV-illuminated) when these ZnO-T are dropped into cell culture medium, they try to acquire an equilibrium stage before their cytotoxic effect starts playing its role. However very controlled experiments would be required to confirm this. As far as the higher toxicity from UV -illuminated ZnO nanoparticles by Zhang et al. is concerned, it could be attributed from different particle-cell interactions between PAA coated ZnO NPs and different culture medium (RPMI 1640) [47].

Other material dependent parameters that have been examined are size, shape and surface area of ZnO structures and their influence on cytotoxicity with respect to NHDF cells. As already demonstrated in Figures 3 and 4, finer ZnO-T exhibit higher cytotoxic effect (EC_50_ [mg/ml] (crushed): 1.0±0.1<(fine): 1.2±0.2<(reference) 3.2±0.3<(thick): 4.5±0.4), which can be easily understood by their larger surface to volume ratio. Our results are in close agreement with the findings with ZnO NP of different sizes towards immune cells [25]. Also Moos et al., have evaluated a higher cytotoxicity in human colon cancer cells in the presence of nanosized ZnO structures as compared to those in micrometer-size regime [43]. However, it is remarkable, that a marginal surface increase of less than 5% for the crushed tetrapods should be responsible for a threefold higher toxic potency compared to the uncrushed material. This leads to the assumption that other mechanisms dominate. For example, it could be that the needle like sharp kinks at broken surface is interacting harmfully with the cell membrane. Another effect might be that as the broken surface areas do not follow high symmetric crystal direction anymore and they exhibit more defects sites and therefore a higher Zn release rate.

Concerning the effect of cell properties on the cytotoxic behaviour of ZnO-T, it has been observed that the primary factor influencing the cell viability, is the passage number of NHDF cells. For experiments with ZnO-T of different ages, we used NHDF with passage numbers until P11. In this range, we observed a high reproducibility (Figure 1). We then carried out experiments with NHDF with higher passage numbers (>P12) to examine the impact of aging effect of ZnO-T on cytotoxicity. Our results indicate the importance of considering the number of preceding cell passages when comparing cytotoxicity experiments. Comparison between cytotoxicities at low (P7–P12) and higher (P14–P19) passage numbers revealed a significant higher sensitivity of the higher passage numbers (Figure 5). Accordingly, Mammone et al., have shown that serial passage of NHDF results in mitochondrial dysfunction and they suggested that apoptotic cell death increases with senescence [48]. Several reports also describe changes in the expression of apoptosis mediated proteins as a function of aging in NHDF, e.g., increase in transglutaminase C in human dermal fibroblasts due to aging [49]. Transglutaminase modifies proteins by cross-linking or polyamination and plays a possible role in apoptosis [50]. Piacentini et al., have described tissue transglutaminase as a “sensitizer” towards apoptotic stimuli specifically targeted to mitochondria [51] and differences between foreskin juvenile and adult fibroblasts with regard to anti-Fas induced apoptosis have been reported by Jelaska et al. [52]. Adult fibroblasts exhibited a significant higher susceptibility to anti-Fas antibody-induced apoptosis than foreskin fibroblasts which has been explained by a process of clonal deletion and expansion with aging [52]. Furthermore Xue et al. have demonstrated the cytogenetic stability of human dermal fibroblasts at a low passage (<P10). In contrast, human dermal fibroblasts at higher passages (≥P10) have been prone to exhibit a higher risk of genetic anomalies [29]. On the basis of the above reports, the higher susceptibility to ZnO-T of long-term cultured NHDF in our study might be explained by alterations in protein expressions or/and activation, possibly facilitating the induction of signalling pathways leading to cell death.

Higher cell densities at constant ZnO-T concentrations result in higher cell viability after 24**h when the passage number of NHDF is below P22 (Figure 6). This observation is in agreement with the published study by Heng et al., in which a higher cell density reduced cytotoxicity compared to lower cell densities in the presence of same ZnO NP concentration [26]. The reason for this could be the reduced ZnO mass per cell or/and a reduced proliferation rate due to contact inhibition as it has been described that ZnO nanoparticles exhibit a selective lethal effect on rapidly proliferating cells [13]. However, NHDF with passage numbers over P22 showed an opposite effect after ZnO-T treatment. As described in Figure 8, NHDF with passage numbers over P22 at high cell density (100000 cells/cm^2^) is significantly more sensitive to ZnO-T than NHDF with passage numbers<P22 (EC_50_ [mg/ml]: P22–P26: 0.5675±0.08281 vs. P11–P14: >10). In contrast, a low cell density (25000 cells/cm^2^) of NHDF with passage numbers over P22 even showed a slightly increased (though not statistically significant) cell viability compared to NHDF with low passage numbers and the same cell density (EC_50_ [mg/ml]: P22–P26: 3.321±0.521 vs. P11–P14: 1.917±0.2716) (Figure 7). Similar tendency has been found in the presence of ZnCl_2_ (Figures 7 and 8). Since the cell density plays a crucial role for the toxicity, we always determined the protein content of untreated NHDF to rule out that this was only an effect of different cell densities due to changes in the proliferation rate. As shown in Figure 8A and Figure 8D, the protein contents of NHDF with low and high passage numbers were comparable. To the best of our literature knowledge this observation has not been described so far and at the moment we can only speculate about the underlying mechanisms. Due to the fact that the sensitivity to ZnO-T drastically increased only in NHDF with high passage numbers (>P22), it is obviously a change in terms of aging which seems to be the case, furthermore it is only relevant for higher cell densities. A possible reason could be on the level of cell-cell contacts and/or cell-cell communication which normally increase in high cell density cultures and result in contact inhibition of further proliferation mediated by inter alia, p38 mitogen activated protein kinase and cyclin-dependent kinase inhibitor p27Kip1 and all of them are also associated with apoptosis [53]–[58]. Furthermore, the cell-cell communication through gap junctions increases in high cell density cultures. Possibly, the ZnO-T/ZnCl_2_ induced death signal in old NHDF cells passes through gap junctions to neighbouring cells (Bystander effect). Thus the sensitivity towards ZnO-T and ZnCl_2_ in old NHDF cells increases in high cell density cultures and vice versa decreases in low cell density cultures. It has been shown that gap junctional intercellular communication (GJIC) can influence the response of cancer cells to cancer chemotherapy agents and a cell density dependent increased cytotoxicity of cisplatin was described by Tong and colleagues which was declared to the greater opportunity for formation of gap junctions between the cells [59].

ZnO structures used in this study exhibit tetrapod geometry with dimensions in the nano-micro scale range and therefore exhibit a larger surface to volume ratio. Comparing cytotoxicity of ZnO-T with ZnO NP, the cytotoxic potency of ZnO-T (EC_50_ = 6.1±0.6 mg/ml) is clearly lower than those of ZnO NP (EC_50_ = 0.022±0.001 mg/ml). EC_50_ values in a similar range have been reported in other studies, e.g., by Heng et al., and also by Ahamed et al., (EC_50_ = in the range of 0.02–0.05 mg/ml) for spherical ZnO NP [26], [60]. The cytotoxic concentration range of ZnO NP is close to the cytotoxic concentration range of ZnCl_2_ (Tab. 7). Deng and Song et el., have also reported the similar cytotoxicity behaviours from ZnO NP and ZnCl_2_ [40], [42]. Several studies analyzing the cytotoxic mechanism of ZnO NP have shown the high impact of dissolved Zn^2+^ ions in the culture medium or inside cells [39], [40], [42]. Also our results suggest that the release of Zn^2+^ ions from ZnO-T at higher concentrations is an important mechanism for cell toxicity (Figure 10). Thus, the clear difference between ZnO NP and ZnO-T (Figure 9) might be caused by the smaller size and higher surface area of ZnO NP (4.7 vs. 40.0 m^2^/cm^3^) and the ensuing increase in Zn^2+^ ions dissociation. Other studies have demonstrated that the particulate matter in contact with the cells leads to cell death [28], [43]. In contrast to ZnO NP, our ZnO-T cannot be taken up by normal cells due to their large biologically effective diameter and thus, are not able to exhibit directly intracellular cytotoxic effects.

We have also addressed the role of Zn^2+^ for cytotoxicity indirectly in the last experiment and the result is shown in Figure 10. It has been shown that by taking supernatants of medium-incubated tetrapods for cell treatment that cytotoxicity obviously also can be induced via released Zn^2+^ alone at a higher dose (20 mg/ml, Figure 10 G and H: 'ZnO-T (S) – disp.'). To clarify if the presence of cells increases this toxicity, e.g., by increasing the solubility of the tetrapods due to lowering the pH, further experiments with transwells (Figure 10 E, F 'ZnO-T in transwells') and others in which we pre-incubated ZnO-tetrapods in the presence of fibroblasts and used the supernatants to treat fibroblasts growing in different culture plates (Figure 10, C, D: 'ZnO-T (S) - disp. + cells') were performed. Nevertheless, they did not result in an increased toxicity of ZnO-T. On the first sight it seems to implicate that cellular factors do not change the toxicity of ZnO-T but it has to be considered that in *in-vitro* systems, one deals with a huge cell to medium volume ratio. Thus, even strong concentration changes of ions or other released products within the nearer cell milieu will be highly diluted by the surrounding medium possibly masking relevant concentrations at the cell surface. Our observations indicate a significant influence of local factors for the toxicity of ZnO-T as ZnO-T directly incubated on fibroblast show a higher toxic potential than in the indirect models (Figure 10). This can be due to Zn^2+^ toxicity, oxidative stress, effects of direct (physical) cell contact and/or other factors. As ZnO-T in present experiments are too large to be taken up by the cells or even the lysosomal compartment, the extremely low pH in the lysosomal compartment is not in focus for the interpretation of our data. It is, an advantage of our model compared to conventional low-size nanomaterials [24], that ZnO-T, though having nano-properties, are not taken up by fibroblasts. This facilitates the understanding of the system, as direct intracellular mechanisms of action do not need to be considered when analyzing the relevant factors for ZnO-T toxicity of that size.

## Conclusions

In conclusion, our results demonstrate that cell culture conditions as well as material properties can change the toxic potency of zinc oxide tetrapods in human dermal fibroblast cell cultures. The impact of different cell densities on the cytotoxicity of ZnO-T is an important aspect when *in vitro* toxicity studies shall be either compared with other *in vitro* studies or when *in vivo* extrapolations are intended. The impact of preceding fibroblast divisions on ZnO-T′s toxicity indicates that the age of a target tissue might be a considerable biocompatibility aspect when developing ZnO tetrapods for biomedical applications. Furthermore, our findings demonstrate that changes in ZnO-T properties by aging up to several months, and by oxygen or UV-light exposure do not impair their biocompatibility, at least in our model. However, other properties like the particle morphology and size involving, e.g., changes of the surface area play a significant role. As the toxic potency already increases when ZnO-T are crushed with a negligible increase of the surface area, the integrity of the nanomaterial is another important aspect, too, and has to be considered concerning human applications. It has also been demonstrated that the ZnO-T used in present study are much more biocompatible compared to spherical ZnO NP. Finally, our results indicate that acute toxic effects of ZnO-T are rather a consequence of local mechanisms (either ascribed to direct cell-particle contact or low distance ionic-effects) than of higher distant mechanisms because the cytotoxicity of zinc ions released from ZnO-T with no cell contact, was much lower than that of ZnO-T having direct cell contact. Taken together, several important cell culture and material parameters have been identified that should be considered for further *in vitro* experiments, hazard assessment, uncertainty analysis and the development of zinc oxide nano-microstructures for different applications.

## Materials and Methods

### Synthesis of nano-micro ZnO tetrapods (ZnO-T)

The freestanding tetrapod shaped ZnO structures with varying size were synthesized by simple flame transport synthesis (FTS) technique [14], [15]. In the FTS process, commercially available Zn powder (diameter ∼5 µm from GoodFellow, UK) and polyvinyl butyral (PVB) powder (Mowital B 60H from Kuraray GmbH, Europe) are mixed in the ratio of 1:2 and the mixture is then heated in a simple box furnace at 900°C for different times ranging from 30 minutes to 2 hours depending upon the requirements. After the process, the snowflake type fluffy material was harvested from the oven which was further investigated to confirm the shape and structures. The growth mechanism of ZnO-T structure has already been described in detail in literature [16]–[18] and same has been used here. The tetrapods are loosely bound to each other and can be separated easily by simply shaking or dispersion, which can be used for biological tests. Batches were synthesized weekly and reproducibility was controlled by phase contrast and scanning electron microscopy (SEM) investigations before cytotoxicity experiments were performed. To determine the total surface area BET measurements of these ZnO-T powders (0.21 g) at saturated N_2_ vapor pressure of 100.2 kPa were performed (BEL Japan, Inc. BELSORP-max). The surface area corresponding to the tetrapods powder specimen was ∼0.84 m^2^/g, assuming the bulk density of ZnO of 5.61 g/cm^3^ this equals 4.71 m^2^/cm^3^. For the calculation of the unit m^2^/cm^3^, which is often used to describe the surface area of nanoparticles, the bulk density has to be considered as ZnO-T exhibit a fluffy material with varying densities.

### Oxygen pre-treatment and UV illumination of ZnO-T

As already mentioned that oxygen vacancies in ZnO structures might influence their cytotoxic behavior, we varied the oxygen vacancy content in these tetrapods. A decrease of oxygen vacancies can be achieved by heating the tetrapods in an oxygen rich environment or the inverse can be achieved by illuminating them with UV light. Therefore, ZnO-T powder (from the same batch) was divided into two parts. One part was treated with oxygen in a tube furnace with a gas (Ar:O_2_:: 80∶20) flow rate of 100 SSCM at 300°C for 1 hour to reduce the oxygen vacancies. The other part was illuminated with UV lamp to increase the oxygen vacancies. For UV illumination ZnO particles were distributed in a plastic petri dish followed by exposure to UV light (254 nm wavelength) over night. As the oxygen vacancy content affects the hydrophilicity of ZnO-T, it also has a strong influence on their wetting behavior. Normal ZnO-T are only slightly hydrophobic however if they are heated in an oxygen rich environment, they become more hydrophobic. Vice versa, when the tetrapods were illuminated by UV light, they turned to superhydrophilic. The hydrophobic and hydrophilic behaviors of ZnO-T under different treatments were confirmed by water droplet tests using contact angle measurement setup.

### Crushed ZnO rods

To investigate the effects from particle geometry and surface on cytotoxicity, crushing of ZnO-T was performed for 2 minutes by a hand mortar and pestle which results in formation of ZnO rods with increased surface area. During crushing the arms of tetrapods get broken and the crushed powder almost exhibits 1D ZnO rod structures. Each broken rod contributes to an additional increase in surface area equal to the two hexagonal area cross-sections at the broken end. Nevertheless, even assuming that all 4 arms break, an increase in surface area would be less than 5%. Also BET measurements could not confirm an increased surface after crushing. Crushed powder even revealed a slight decrease in the total surface area as compared to that of tetrapods for the same probe, possibly, because in the specimen chamber these 1D structures in the crushed powder were lying over each other preventing nitrogen gas to be adsorbed completely to the particle surface.

### Spherical ZnO nanoparticles (ZnO NP)

To understand the shape dependence of ZnO-T induced cytotoxicity, spherical ZnO NP, which are used most commonly in cytotoxicity studies, were used as reference material. Spherical ZnO NP (average diameter ∼150 nm, calculated surface area 7.1 m^2^/g, based on the bulk density this equals 40.0 m^2^/cm^3^) were prepared according to the Leidenfrost drop method as described before [61], [62].

### Scanning electron microscopy (SEM)

The morphology of snowflake type material harvested from the FTS process was investigated by scanning electron microscope (Philips XL-30 microscope) equipped with LaB_6_ filament and energy dispersive X-ray diffraction analysis (EDAX) detector. SEM images were recorded at 10 kV acceleration voltage with 20 µA beam current at the University of Kiel. SEM investigations confirmed that this snowflake type material consists of tetrapod shaped ZnO structures. The thicknesses of the tetrapod arms vary from 200 nm to 2 µm. Their lengths are in the range from 500 nm to 50 µm. The crushed powder was also investigated inside SEM which confirmed that during crushing the arms of tetrapods are broken and thus forming 1D rods.

### Cell cultures

Normal human dermal fibroblasts (NHDF) (PromoCell, C12300) were cultured in Quantum 333 (PAA, U15-813) supplemented with 1% (v/v) penicillin/streptomycin (PAA, P11–010). NHDF are human primary cells which were isolated from the dermis of juvenile foreskin or adult skin with different origins (PromoCell). For stock cultures, 750000 cells in 20 ml medium were seeded weekly into a T75 flask (Sarstedt) and kept at 37°C and 5% CO_2_ in a humidified atmosphere. The medium was changed every two to three days. For sub-culturing, cells were trypsinized by Trypsin/EDTA (PAA, L11-659). Cells were counted under the microscope.

### Phase contrast microscopy

Morphological changes of NHDF cultures and also ZnO-T (in clear bottom microtiter or 6-well plates) were examined in parallel to the performed cytotoxicity tests by means of an inverted phase contrast microscope (IMT-2, Olympus, Hamburg, Germany) equipped with a digital camera (E-300, Olympus, Hamburg, Germany).

### Sample preparation

Powder of ZnO-T was scaled and brought for each experiment fresh into dispersion with culture medium at an initial concentration of 10 mg/ml. One reproducible standard batch of ZnO-T served as reference in all experiments and was set in relation to other samples. As ZnO-T particles settled within a short time, the dispersion was shaken by a vortexer (Reax Control, Heidolph) before each sampling. Powder of spherical ZnO NP was brought into dispersion with culture medium at an initial concentration of 10 mg/ml. The ZnO NP dispersion was ultrasonicated for 1.5 h and shaken by a vortexer to achieve homogeneity before a serial dilution over a concentration range from 35 to 15 µg/ml was prepared in culture medium. ZnCl_2_ was purchased from Merck (No. 108816). The stock solution (5 mg/ml) was prepared in aqua bidest and then sterile filtrated. A serial dilution over a concentration range from 40 to 25 µg/ml was made in culture medium.

### Determination of cell viability by the MTT assay

The MTT assay was used to estimate the viability of cells by measuring the production of formazan mainly through the activity of cellular mitochondrial dehydrogenase. Cell viability was determined accordingly as described in already published paper [63], [64]. The sample was replaced with 312.5 µl/cm^2^ fresh culture medium per well. In brief, MTT [3-(4,5-dimethylthiazol-2-yl)-2,5-diphenyltetrazolium bromide] solution (1 mg/ml medium) was added to each well and incubated for 90 minutes at 37°C and 5% CO_2_. Subsequently 312.5 µl/cm^2^ solubilization solution (20% (w/v) SDS, 2.5% (v/v) 1NHCl and 2.5% (v/v) acetic acid (80%) in 50% (v/v) DMF, pH 2) was pipetted to each well. Production of formazan by viable cells was estimated after 90 min (37°C, 5% CO_2_) by measuring the absorbance at a wavelength of 570 nm (photometer 340 ATTC, SLT Lab instruments, Germany). Before, 180 µl of the sample/supernatant were transferred into a new 96-well plate since ZnO-T at higher concentrations form thick white sediments causing interferences with the measurement. Also 180 µl of untreated samples or samples treated with conditioned medium were transferred into new 96-well plates before measuring to ensure a uniform treatment and adequate control. As further control we performed at first experiments without cells to exclude a direct catalytic effect of ZnO-T on formazan formation. Absorbance measured by means of the photometric assay did not differ from background values of the assay (data not shown). Additionally, in each experiment phase-contrast microscopy was performed in parallel for each treatment group to verify photometric with morphologic changes in the cell culture.

### Determination of total cell protein by the Lowry assay

Total protein concentrations of controls were determined by the Lowry assay [65] for each experiment as parameter for the final cell density used for treatment with ZnO-T, ZnCl_2_ or ZnO NP. This was done to reassure the same initial cell concentrations as the toxic potency of a substance can depend on this parameter. Therefore, cells were washed three times with phosphate-buffered saline (PBS) and incubated for 45 min with 0.5 N NaOH. The protein amount was measured by colorimetric determination at 620 nm (photometer 340 ATTC, SLT Lab instruments). Bovine serum albumin was used as standard.

### Cytotoxicity of ZnO-T with different ages

NHDF cells (passage numbers: P7 –P11) were seeded into clear flat 96-well microtiter plates (Falcon®, 3072) at a cell density of 50.000 cells/cm^2^. After two days in culture, the cells were exposed to ZnO-T, which exhibited the same morphology but were synthesized at different dates. Ages of ZnO-T used for cell treatment were: 16–20 weeks, 13–17 weeks, 8–12 weeks and 3–7 weeks. Following measurements were performed 24 h later.

### Cytotoxicity of ZnO-T with different surface charges

NHDF cells (passage numbers: P8–P20) were seeded into clear flat 96-well microtiter plates (Falcon®, 3072) at a cell density of 50.000 cells/cm^2^. After two days in culture, the cells were exposed to ZnO-T (reference), ZnO-T (UV-treated) and ZnO-T (O_2_-treated). Cell viability was analysed 24 h later.

### Cytotoxicity of ZnO-T with different morphologies

NHDF cells (passage numbers: P10–P14) were seeded into clear flat 96-well microtiter plates (Falcon® 3072) at a cell density of 50.000 cells/cm^2^. After two days, the culture medium was removed and cells were exposed to varying concentrations of ZnO-T with different morphologies: ZnO-T (reference), ZnO-T (thick), ZnO-T (fine) and ZnO-T (crushed). Cell viability was analysed 24 h later. The cytotoxicity of ZnO-T (reference) was set in relation to other ZnO-T samples with varying morphologies.

### Cytotoxicity depending on cellular age

Thawed NHDF cells had the passage number P5 and were cultivated for at least one week before they were used in experiments. Cells were weekly passaged, this means one passage number implies a time period of one week. Short-term cultivated NHDF cells (passage numbers: P7–P12) and long-term cultivated NHDF cells (passage numbers: P14–P19) were seeded at a cell density of 50000 cells/cm^2^ in 312.5 µl medium/cm^2^ into clear flat 96-well microtiter plates. After two days, the cells were treated with different concentrations of ZnO-T (reference). Cell viability was analyzed 24 h later.

### Cell density assay

NHDF cells (passage numbers: P11–P15) were seeded into clear flat 96-well microtiter plates (Falcon®, 3072) at different densities of 25000, 50000 and 100000 cells/cm^2^ in 312.5 µl medium/cm^2^. After two days in culture the culture medium was completely removed and replaced by medium with varying concentration of ZnO-T (reference) or ZnCl_2_. Following measurements were performed 24 h later.

### Cell passage and cell density assay

Short-term cultivated NHDF cells (passage numbers: P11–P15) and long-term cultivated NHDF cells (passage number P22–P26) were seeded at two different cell densities of 25000 and 100000 cells/cm^2^ in 312.5 µl medium/cm^2^ into clear flat 96-well microtiter plates. After two days the cells were treated with different concentrations of ZnO-T (reference) or ZnCl_2_. Following measurements were performed 24 h later. The experiment was additionally repeated with three different batches of NHDF cells.

### Cytotoxicity of ZnO-T without direct cell contact

NHDF cells (passage numbers: P12–P14) were seeded at a cell density of 50000 cells/cm^2^ in 312.5 µl medium/cm^2^ into 6-well plates (Sarstedt, 83.1839). After two days, cells were treated with ZnO-T (reference), ZnO-T (reference) in transwells (Costar, 3 µm pore size, 3418) positioned on top of NHDF cells as wells as supernatant of ZnO-T (reference). Supernatants of ZnO-T (reference) were prepared as follows:

‘ZnO-T (reference) were dispersed in two different concentrations (10 mg/ml and 20 mg/ml) in culture medium. The dispersions were pre-incubated with or without NHDF cells (50000 cells/cm^2^ into 6-well plates) for 24 h at 37°C and 5% CO_2_. Then, ZnO-T dispersion was collected in 15 ml tubes and centrifuged at 600× g for 10 min. The supernatants were used to treat NHDF cells. After 24 h the viability was measured by the MTT assay.'

### Statistical analysis

At least three determinations with at least four wells per test compound concentration for the cytotoxicity assays were carried out with cultures of different sub-cultivations. Mean values of determinations from different cultures and the standard errors (SE) of the mean were calculated and shown in the diagrams (Figures 2, 4, and 5). Statistical significance was determined by one-way analysis of variance. Significance was ascribed at p<0.05 (*); p<0.01 (**) and p<0.001 (***). All analyses were performed using the program InStat3. Median effective concentrations (EC_50_ values) were determined by using the software *GraphPad Prism* version 4.01 for Windows (GraphPad Software, San Diego California USA, www.graphpad.com). Curve fitting was done by nonlinear regression analysis using the Hill equation: 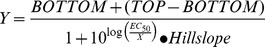. Constraints: BOTTOM = 0 and TOP = 100.
